# Hybrid fusion of E-nose and computer vision using optimized deep learning and machine learning for robust plant leaf recognition

**DOI:** 10.1038/s41598-026-58222-6

**Published:** 2026-06-15

**Authors:** Pouya Bohlol, Mohammad Hasan Sabet Dizavandi, Syed Saeid Mohtasebi, Mahmoud Omid

**Affiliations:** Department of Agricultural Machinery Engineering, Faculty of Agricultural Engineering and Technology, University of Tehran, Karaj, Iran

**Keywords:** EfficientNetB7, Big data set, Multisensory system, Late fusion model, Optimization, Computational biology and bioinformatics, Engineering, Mathematics and computing, Plant sciences

## Abstract

The fusion multi-sensory system with optimized deep learning and machine learning algorithms appeared to synergize difficult paradigms in precision agriculture and boost recognition of various plant species. In this study, an electronic nose (E-nose) system with eight MOS sensors and a computer vision platform were designed for constructing a large-scale data set of 26 various species of plant leaves collected during 2 years under different environmental conditions. After preprocessing functions, the E-nose data set consisted of 15,600 data points, while two-phase data augmentation expanded the image data set to 156,000 samples. Multiple machine learning models, including MLPs and 7 machine learning algorithms, were executed and optimized based on the E-nose dataset, where KNN with 97% accuracy and 5 ms response time per sample showed the highest accuracy in recognizing leaves. In parallel, six deep learning and vision transform algorithms were implemented, and hyperparameters such as batch size, learning rate, optimizer, and image size were updated during training. InceptionV3 demonstrated superior performance compared to others, with 99% accuracy, 0.2 loss, and 8 ms response time. Finally, a fusion model of E-nose and computer vision-based deep and machine learning algorithms was developed for robust performance of recognizing 26 different species of leaves. The fusion model could recognize leaves with 99.8% accuracy with a 10-ms response time. The proposed hybrid approach highlights the potential of multi-modal fusion for reliable, fast, and scalable solutions in smart agriculture.

## Introduction

Plants are essential to human life and have long been used by indigenous societies as traditional remedies. Practitioners often use their olfactory or sensory skills, which have been honed over years of practice, to identify therapeutic plants^1^. Conserving and managing the important and endangered plant species was very important for ecosystems. Understanding species and classifying them through leaves can help track biological changes and prevent species extinction. Recognizing different species or varieties of plants plays an important role in optimizing production and farm management^2^. Plant leaf identification can help farmers identify pests, diseases, and even nutritional needs. Classifying plants through leaves can help quickly identify medicinal species and prevent the misuse of poisonous species. Identifying and monitoring plant species is important for assessing forest health, protecting species diversity, and planning for sustainable use of natural resources^3^.

Approximately 391,000 vascular plant species worldwide; it is impractical for experts to identify and classify them all, especially given the high similarity among some species. Additionally, the threat of extinction highlights the need for proper conservation of endangered plants. Automated systems are essential for efficient plant identification and classification, utilizing features like leaf shape, texture, veins, and color^4^. So the identification of herbs based on scientific data has been greatly aided by recent developments in analytical technology. Many individuals find this comforting, particularly those who are not familiar with herbal identification. In addition to time-consuming methods, laboratory-based testing needs expertise in sample treatment and data interpretation^5^.

Traditional plant monitoring methods, such as field surveys and satellite remote sensing, often require expertise in disciplines like microscopy, chemical analysis, and molecular biology. While these techniques are highly specialized, they can be challenging to execute, prone to biases in assessment, and inefficient for precise species identification^6,7^. However, automated monitoring technologies such as deep learning and electornic tongue and noses can effectively overcome these limitations by enabling large-scale, real-time monitoring of plants^8,9^. Therefore, there is an urgent need to develop efficient, real-time methods for the recognition of aquatic plant species.

The electronic nose (E-nose) is one of the recognition and classifying tools that works based on aroma. The E-nose machine system uses intelligent sensors to simulate the human olfactory system, enabling it to identify volatile gases emitted from agricultural and food products^10^. An electronic nose device is designed to detect and differentiate between complex odors using a series of metal oxide semiconductor sensors. When exposed to an odor, the sensor array generates a unique electronic fingerprint. This response from all the sensors is documented as an electronic fingerprint^11^. Various artificial neural network (ANN) machine learning (ML) algorithms can be applied to these fingerprints to identify different plants.

The swift progression of artificial intelligence and deep learning techniques within the domain of computer vision have achieved substantial advancements across numerous practical applications. These include forest fire detection, agricultural monitoring, and medical diagnostics, highlighting their considerable promise for integration into plant monitoring systems^12,13^. Leaves of plants have different morphology and similar color and texture, and overall similarity points are too much. So a multi-sensory system was designed for multi-object recognition and difficult feature extraction with high accuracy and least loss.

In this study we focus on the recognition method and creating a significant data set. In contrast to the previous method that focused attention on one framework and system for identification, we propose a multi-sensory system. The high similarity point of plant leaves induced challenges in the identification system. A multisensory system was proposed for better performance and recognition of complex patterns. This system consisted of E-nose and computer vision (CV), so both olfactory and visual characteristics of plant leaves could be identified with high accuracy. For this investigation, two data sets were created with an E-nose and a computer vision system. The data set consisted of 20 different leaves of plantscollected from various places. E-nose system data was analyzed with ANN and ML algorithms. The computer vision system extracted visual features for transferring to deep learning algorithms. Combining an E-nose and a computer vision system provides a wide overview of analyzing in multi-spect. These systems and powerful data sets could improve the performance and cover the defect of analyzing. Beyond scientific and environmental benefits, automated plant leaf recognition can provide tangible economic advantages by reducing labor costs associated with manual identification, accelerating agricultural monitoring and post-harvest management, and enabling the development of commercial applications such as mobile or web-based plant recognition tools.

The main contributions of this work are summarized as follows:

- We design and implement a multisensory plant leaf recognition system that jointly utilizes E-nose (olfactory) signals and computer vision (visual) data to address the challenge of high similarity among plant leaves.
- We construct two dedicated datasets (E-nose sensor dataset and leaf image dataset) collected over an extended period and across multiple locations, enabling robust evaluation of multisensory recognition.
- We provide a comprehensive comparison of machine learning classifiers, ANN-based models, and deep learning architectures for plant leaf recognition across both modalities.
- We introduce a late-fusion strategy to combine predictions from olfactory and visual models, improving recognition accuracy compared to single-modality systems.

The rest of this paper is organized as follows: Section two briefly introduces the related work of E-nose, deep learning, convolutional neural networks (CNN), ANN, and ML algorithms. Section three demonstrated the E-nose and computer vision system with the introduction of various ANN, ML algorithms, and deep learning structures. The result of ANN, ML, and deep learning (DL) architectures is shown in section four. Section five demonstrated the conclusion.

## Related work

Today, knowledge and information of plant is important for scientists, marketing and industrial. Automatic recognition of plants is an crucial part of agriculture engineering and postharvest, E-nose, ML and ANN combined to increasing precision in identification and classification. Traditional method and Standalone systems can not work with high performance and accuracy. This section reported the review of different investigation in E-nose, computer vision, machine learning, artificial neural network and deep learning algorithms that reported in Table 1.

**Table 1:** Summery of related work in leaves of plants classification with deep learning and E-nose system.

Site	System	Architecture	Dataset information	Accuracy (%)	References
Medicinal plants	CV & RGB	CNN and Random forest	heterogeneous features	99	Sharma et al.^27^
Rice	CV & RGB	CNN	10 common rice diseases	95.48	Lu et al.^28^
Rice Plant	CV & RGB	CNN with pre-trained VGG16	7 classes of deases	93	Shrivastava et al.^29^
Medicinal Plant	Multispectral and Texture Feature	Random forest	6 classes of medical plant	99	Naeem et al.^30^
Plant leaf	CV	ResNet-50	leaf diseases	99	Reddy et al.^23^
Plant	CV	Optimized ResNet-110	CVIP100 leaf dataset	87	Wu et al.^31^
Plant leaves	CV	ANN&SVM	285 images	93	Sharma et al.^32^
House Plant	CV	ResNet-50 and MobileNet_v2	2500 images	98	Hama et al.^33^
Potato leaf	CV	Transfer learning and DCNN	lethal diseases detection	96	Lanjewar et al.^34^
Tea leaf	CV	DL and ML classifier	leaf diseases	99.5	Panchbhai and Lanjewar^35^
Tea Plant	E-nose—ML	SVM	undamaged, mechanically damaged, damages	100	Sun et al.^15^
Grape Cultivar	E-nose – ML and ANN	ANN	5 Class	99	Khorramifar et al.^36^
Essential Oils	E-nose—ML	SVM	Classification and Identification 6 Classes	100	Rasekh et al.^37^
Tomato Plants	E-nose	PCA	Diagnosis of Aphid-Stressed	86.7	Cui et al.^38^
Plants	E-nose—ANN	radial basis function artificial neural network (RBF-ANN)	Asteraceae family, 8 class	100	Zou et al.^39^
essential oil from *Rosa damascena*	E-nose	LDA^a^	6 parameters of quality	95	Gorji-Chakespari et al.^14^

^a^linear discrimination analysis.

### Electronic nose

The EN is a device that can identify both basic and complicated scents thanks to a variety of electronic gas sensors with partial specificity and a suitable pattern recognition algorithm. Gorji et al. work on the classification of essential oil composition in Rosa damascena Mill genotypes using an electronic nose. Nine genotypes of Rosa were grouped in three classes based on characteristics that GC–MS determined. The En analyzed the classes and could identify by LDA, (linear discrimination analysis.) PCA (principal components analysis.) and SVM (Support vector machine.) algorithms with 85%, 99% and 99%, accuracy. These results reported that EN could identify three quality classes of essential oil with high precision, and this system is inexpensive, fast, and nondestructive^14^. Sun et al. were classifying damage on tea plants with E-nose and ML algorithms. Feature selection was done with PCA, and the damage was detected with MLP and SVM methods with 100% accuracy^15^. Kombo et al. improved the E-nose system and piecewise feature extraction method to classify high-quality black tea. SVM coupled with the piecewise feature method could classify with 96.5% accuracy for test data^16^. Ihsan et al. enhanced E-nose performance and polynomial feature extraction for classifying bean coffee. Four popular machine learning classifiers such as LDA, SVM, LR (Logistic Regression.) and QDA (quadratic discriminant analysis.) were used for quality assessment. E-nose and LDA could improve quality management with 87% accuracy^17^. Hazarika et al. detected the pathogen in oranges with a developed e-nose system. Five ML classifiers were selected for identifying pathogens in 21 plants. Finally, the boosted decision tree could classify the objectives with 99.36% accuracy^18^.

### Machine learning and artificial neural network

ANN and ML are widly used for pattern recognition of detection and anlalyzing systems. Artificial Neural Networks (ANN) are computational models that are based on the architecture and operation of biological neural networks found in the human brain. They are made up of interlinked nodes, or neurons, structured in layers. These networks are particularly effective at tasks like pattern recognition, classification, regression, and decision-making, and they have numerous applications in different fields. Machine learning, which is often referred to as predictive analytics or statistical learning, operates at the crossroads of statistics, artificial intelligence, and computer science. The primary aim of machine learning is to create predictive or descriptive models based on sample data or historical experience to forecast either the value of a continuous output or the category of a classification output. Thiagarajan et al. used CNN (Convolotional neural network.) for extracting features of the plant banana behind ANN and ML algorithms for identification and classification. ANN with the scale-invariant feature transform (SIFT) method could recognize the disease of the banana plant with 95% accuracy^19^. Dahigaonkar et al. use SVM for classification of 128 images of 32 species of medical plants with 96.7% accuracy^20^. Begue et al. used SVM, KNN, (K-nearest neaborhod.) MLP (Multi-layer preceptron.) and RF (Random forest.) to classify 30 images of 20 species of medical plants with 90.1% accuracy^21^. Janani et al. developed ANN algorithms for the identification 63 images of 6 species of Indian medical plants with 94.4% accuracy^22^.

### Deep learning algorithms

Advancements in deep learning have significantly improved plant detection, with convolutional neural networks (CNNs) becoming a popular backbone architecture for researchers. These methods, including hybrid models and deep learning architectures, enhance plant categorization and identification. Raddy et al. designed a multisensory system with red deer and a deep convolutional neural network for plant classification and reducing consumption time of training and response. First, ResNet50 was used for feature extraction, and after that, the modified Red Deer optimization algorithm was used for optimizing the features. Plant disease identification convolutional neural network (PDICnet) achieved 99.73% accuracy for 6 classes of diseases^23^. Razavi et al. detected the quality of rice cultivars by ResNet and the transfer learning model. Three ResNet algorithms were compared with the proposed algorithm that was achieved by improving ResNet50 by changing the extracting features layers. The proposed algorithms could recognize the quality of rice with an average of 98.13% accuracy^24^. Aimen et al. designed an automatic classification plant based on their leaves. After three distinct processes, such as preprocessing, feature extraction, and classification, ANN could recognize the various types of leaves with 96% accuracy. Yigit et al. used an artificial intelligence technique for identifying plant leaves. 32 different plant leaves were used for creating the data set, and SVM algorithms could identify leaves with 94% accuracy^25^. Tavakoli et al. used CNN for classifying 12 different cultivars of common beans that belong to three various species. They used Additive Angular Margin Loss and Large Margin Cosine Loss instead of softmax, and the accuracy of classification increased to 95.86% accuracy^26^. 

**Fig. 1:**
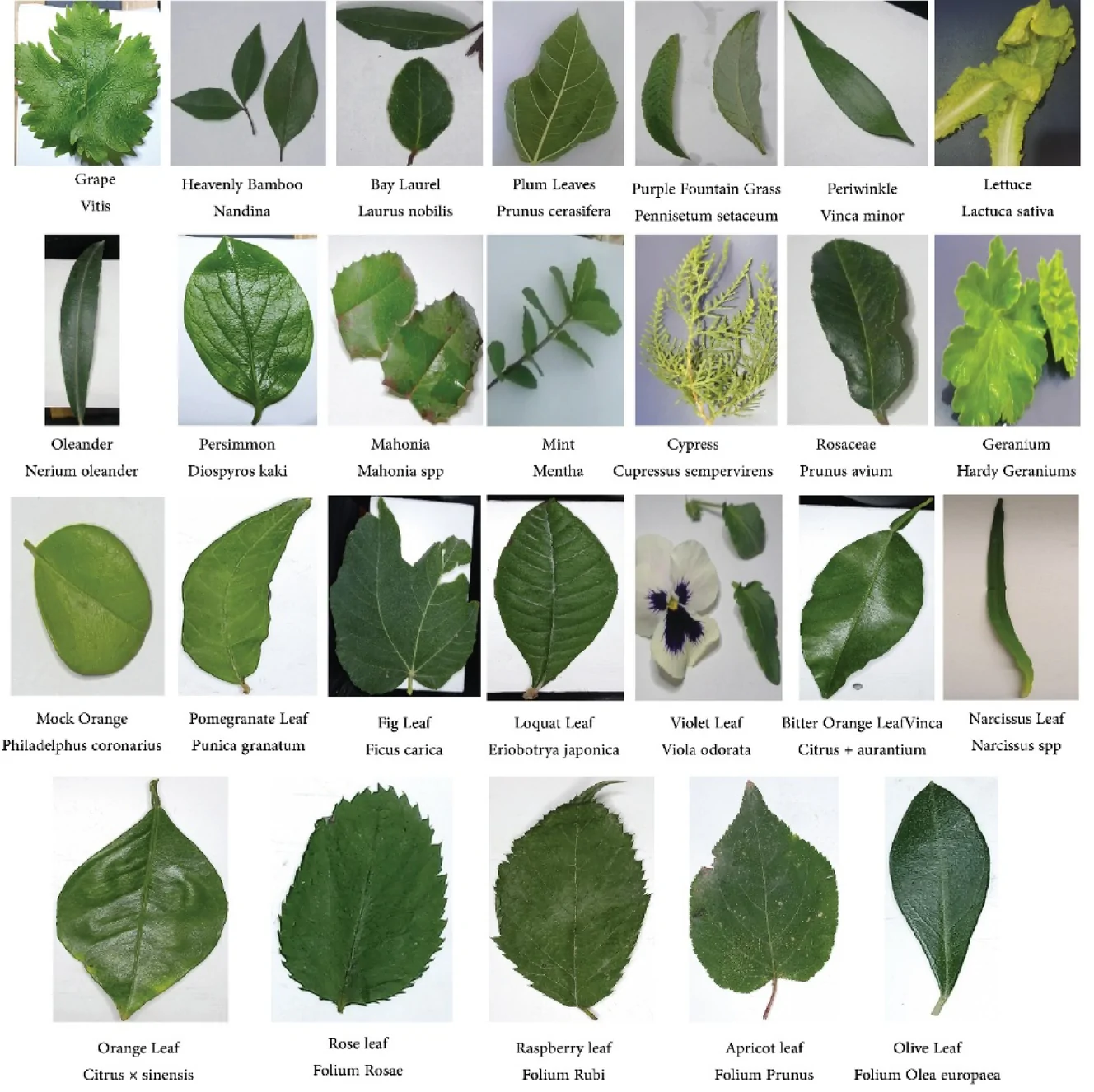
Information leaves of plant that selected with specific situation in multi season.

Although multimodal fusion of electronic nose and computer vision has been explored in other domains such as food quality assessment and geographic origin classification, these studies have focused on quality, adulteration, or regional discrimination tasks rather than plant leaf species recognition. For example, prior work has fused E‑nose and vision for predicting kiwifruit attributes and tea quality, demonstrating improved classification performance with fusion strategies. To the best of our knowledge, no prior study has integrated electronic nose and computer vision modalities specifically for plant leaf recognition across a real, multi‑species dataset covering 26 plant classes. Moreover, the proposed framework combines these modalities with both machine learning and deep learning models and a decision‑level fusion strategy to form a comprehensive multisensory recognition system, which has not been implemented previously in leaf identification literature (Fig. 1).

## Material

### Samples

**Fig. 2:**
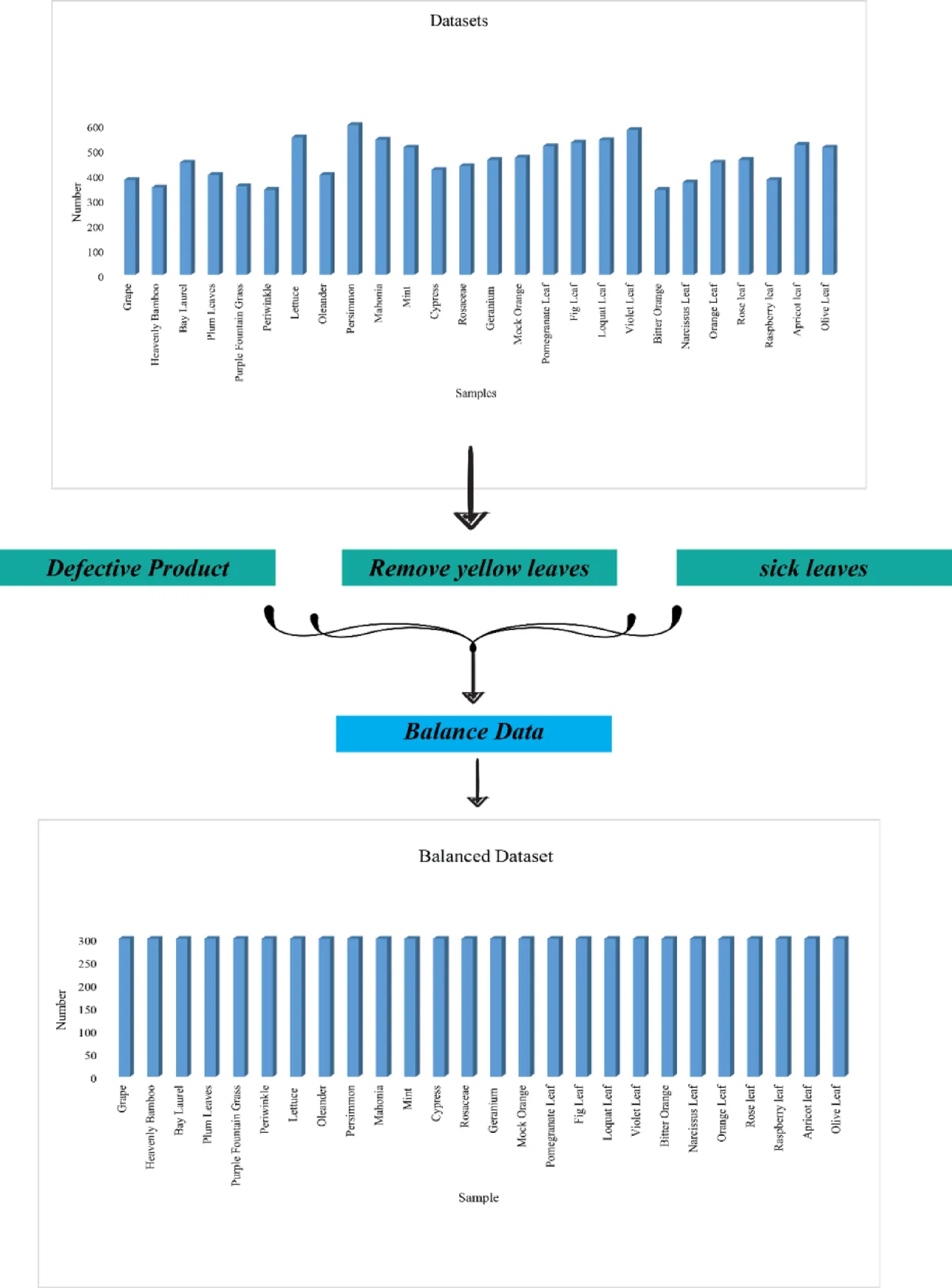
Showed Processing of Blancing data set and number of samples in each classes.

The plant dataset consisted of 26 distinct plant leaf categories collected from various locations. For each location, leaves were sampled from 2–3 trees, and collections were performed across different seasons over two years to increase diversity. Leaves were collected from different parts of the trees (varying heights and sizes). Selection criteria for each leaf included being green in color and free from visible damage. In total, 11,857 leaf samples were collected. During dataset screening, some leaves exhibited abnormalities such as black spots, physical damage, or irregular shapes. These low-quality samples were excluded to ensure dataset consistency and reliability. After refinement, the final dataset contained 7,800 high-quality leaf samples representing all 26 classes, as shown in Fig. 2, which illustrates the dataset cleaning procedure.

### Electronic nose systems

**Fig. 3:**
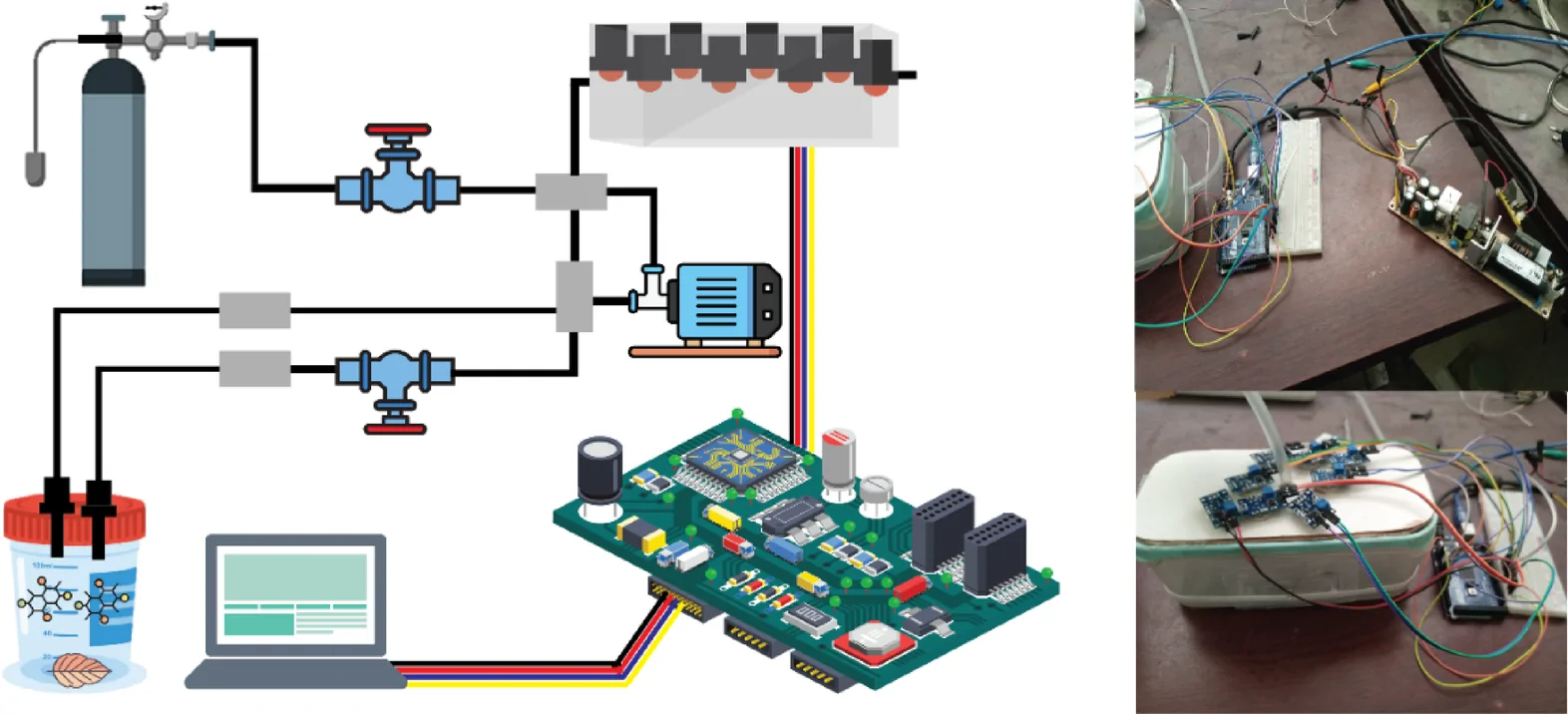
shows a schematic diagram of an artificial olfactory (E-nose) sensory system. This system includes several components, such as a sample container, micropump, air filter, gas sensor array, Arduino board, and computer system.

**Fig. 4:**
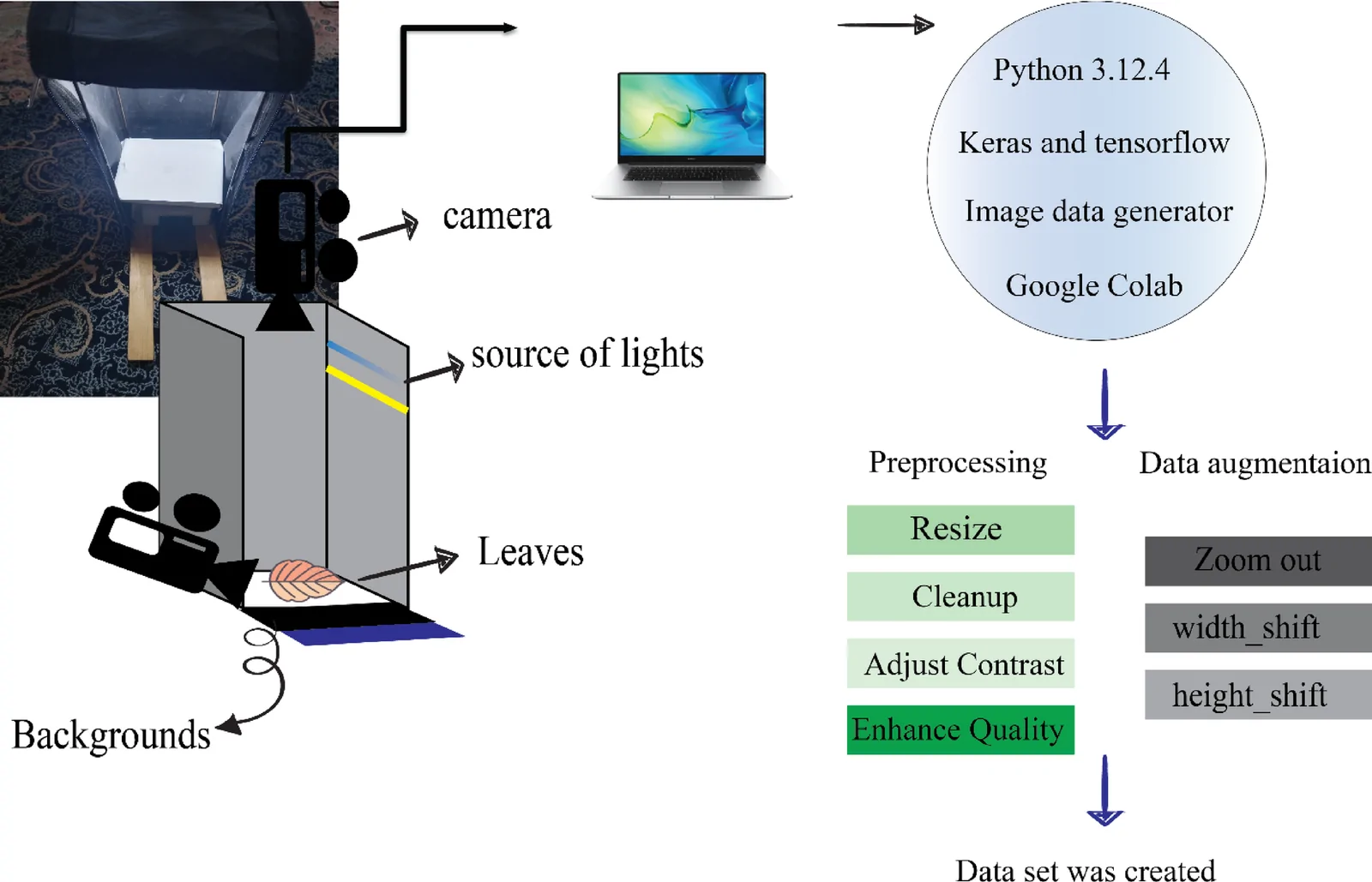
Platform of computer vision system for Image aqusations.

Extracting fingerprints of odors was done with an electronic nose system based on metal oxide semiconductor (MOS) sensors. The developed E-nose system consists of a sample chamber equipped with sensors, a diaphragm pump, a power supply, a carbon filter, and a data acquisition board. A schematic demonstrated the E-nose and measurement system component in Fig. 3. This system constructed eight tin oxide-based gas sensors from Hanwei Electronics Co., Ltd., located in Henan, China, with each sensor reacting most strongly to a specific odor (Fig. 4). The efficacy and precision of the system are significantly contingent upon the judicious selection of sensors. Comprehensive specifications of the sensors utilized in this research are delineated in Table 2.

**Table 2:** MOS gas sensors used in E-nose with their specifications.

Number	Sensor	Target gas	Detection range (ppm)
1	TGS822	Alcohol, Ethanol, Organic Gases	50–5000
2	TGS2610	Butane, LPG	1–25
3	TGS2602	Air Pollutants	1–30
4	TGS2620	Alcohol, Organic Gases	50–5000
5	MQ8	Hydrogen	1000–100
6	MQ5	Natural Gasses, LPG, Coal gas	1000–100
7	MQ138	Hexane, Benzene, NH3, Alcohol, CO, Smoke	500–5
8	MQ135	Alcohol, Benzene, Smoke, CO, CO2	300–10

### Computer vision system

**Fig. 5:**
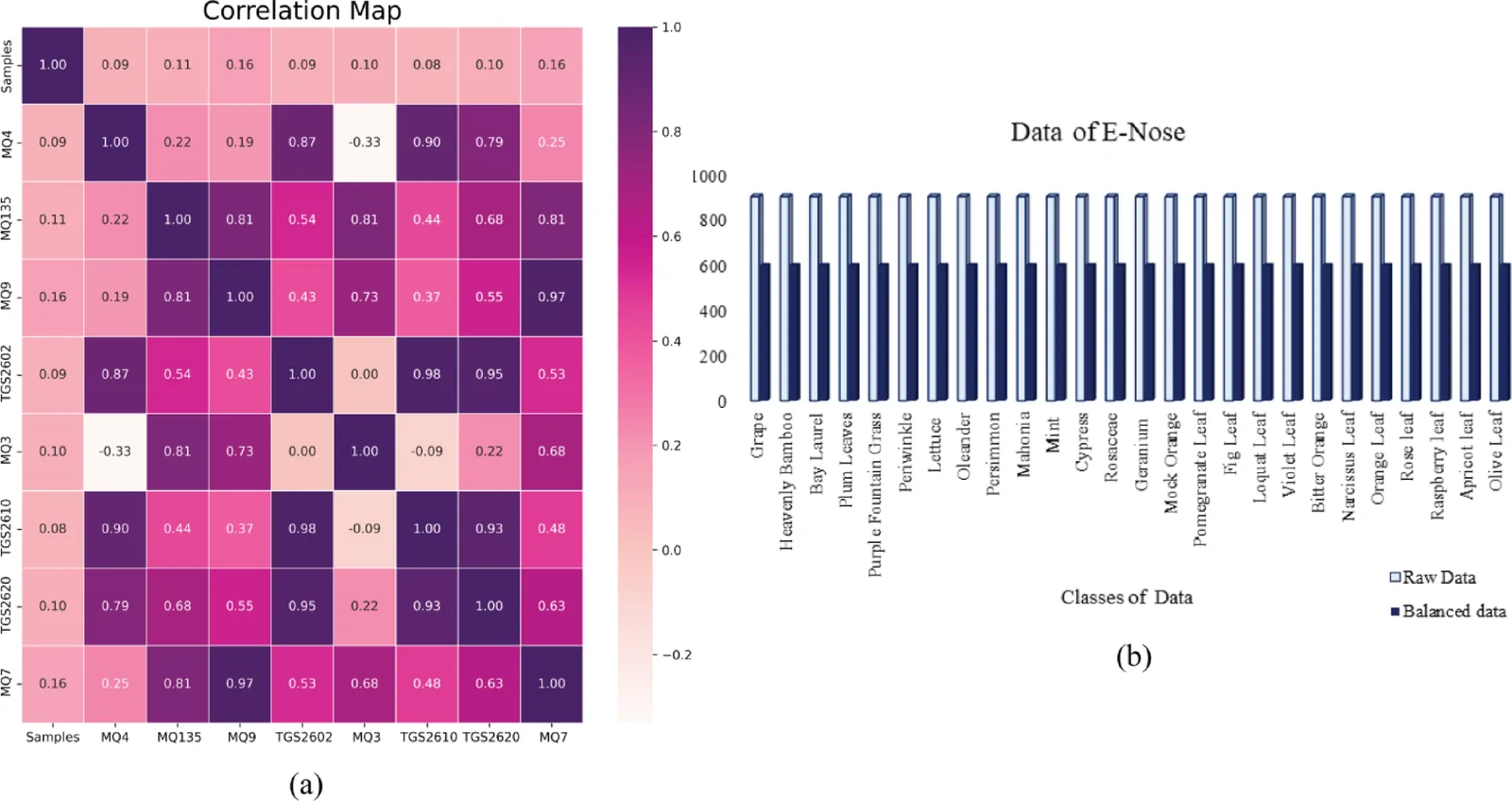
illustrates the E-nose data set and the correlation of sensors and samples. (**a**); The heatmap demonstrated the correlation between 8 sensors after feature extraction and samples. (**b**); the number of data points that the E-nose system extracted before and after processing.

Detecting plant leaves using only an electronic nose (E-nose) has its challenges; therefore, a computer vision system was developed to enhance the information gathered about plant leaves. The computer vision system consists of a camera, an imaging box, illumination, and a background, as shown in Fig. 5. This system utilizes multiple backgrounds, different cameras, and various imaging angles.

### Pre-processing dataset

The preprocessing of data collection was conducted in two steps. In the first step, features were extracted from sensor data using significant preprocessing methods. The aim of data preprocessing in the E-nose system is to select important features from the sensor responses and transfer them to pattern recognition algorithms. The selection of the best features through preprocessing was performed in three steps: baseline manipulation, compression, and normalization. In the initial phase of the analysis, the sensor responses were subjected to manipulation in relation to their baseline values. This was conducted to achieve drift compensation, enhance contrast, and implement scaling through a fractional method^40^. Specifically, in this approach, the baseline is subtracted from the sensor response, followed by division of the resultant value by the original baseline according to Eq. 1. The second stage involved feature extraction from sensor responses during the injection phase, as discussed by Pearce, et al.^40^. This process compresses sensor response data, leading to enhanced selectivity, reduced acquisition time, and an extended lifespan for the sensors^41^. Normalization is commonly employed to eliminate the scaling effects caused by concentration variations on sensor patterns^42^. Among the various scaling techniques, auto-scaling is the most widely used, particularly when sensor responses are measured on different magnitude scales^43^. This method standardizes variables as outlined in Eq. 2. After implementing all functions and the third iteration in each sampling, data set of E-nose constructed 600 samples per class for all 26 classes (totaling 15,600 samples) that is shown in Fig. 5b. The correlation among sensing channels after post-extraction is shown in Fig. 5a. To ensure a fair comparison among all machine learning, ANN, and fusion models, the E-nose dataset was divided using a fixed and identical evaluation protocol. Specifically, samples from all 26 classes were randomly split into training, validation, and testing subsets with the same ratio used for the imaging dataset. The split was performed in a stratified manner to preserve class balance, and the same train and test partitions were applied consistently across all E-nose-based models and the fusion framework.

1 $${y}_{s}\left(t\right)=\frac{{x}_{s}\left(t\right)-{x}_{s}\left(0\right)}{{x}_{s}\left(0\right)}$$

2 $${z}_{j}=\frac{{x}_{j}-{x}_{l}}{{st}_{l}}$$

**Fig. 6:**
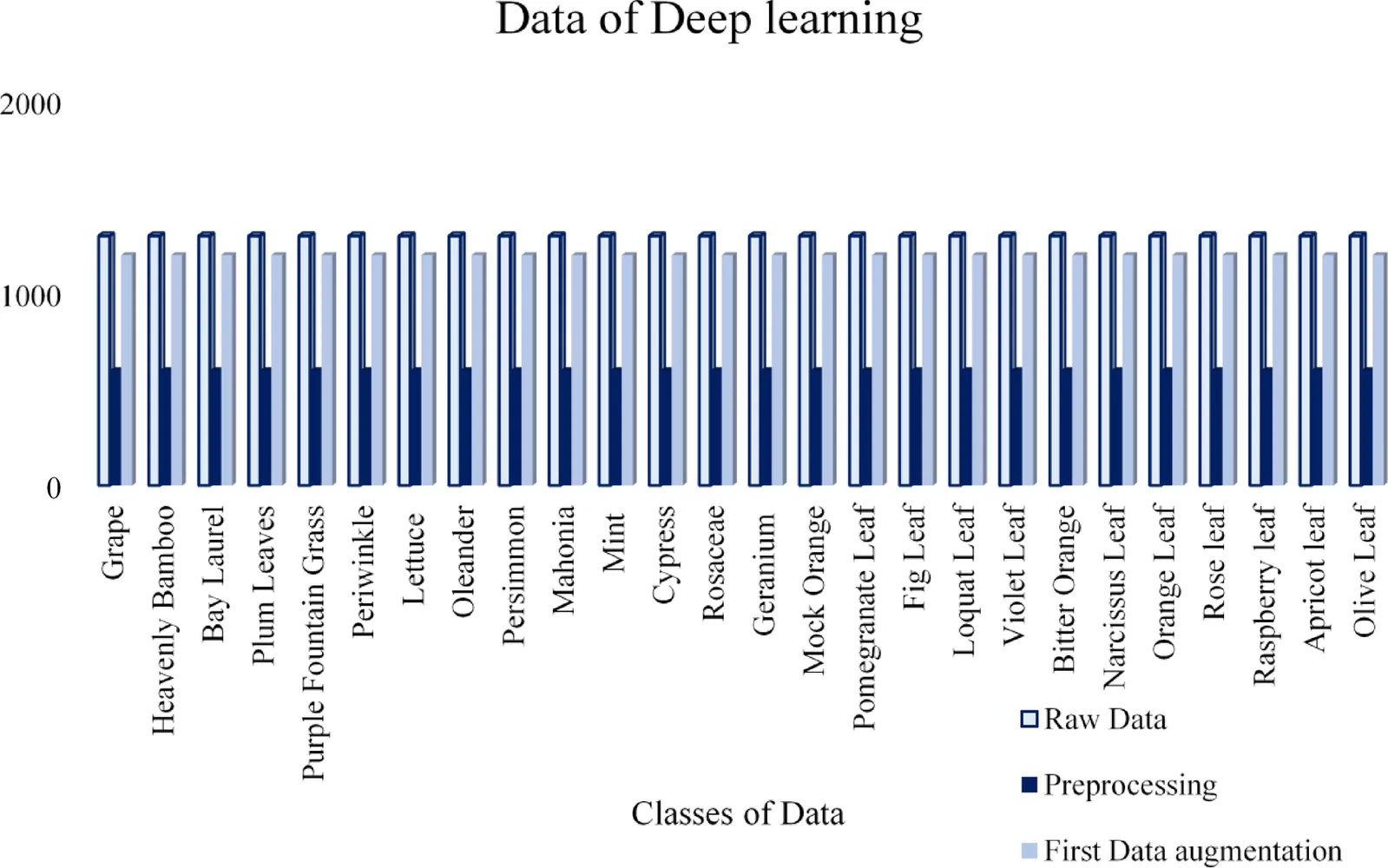
Illustrates the number of data points that the computer vision captured before and after processing for deep learning architectures.

In step two, computer vision data were processed in various stages. The preprocessing involved adjusting the quality, resolution, and contrast of the images, reducing noise, and removing missing data. This methodological framework enhances the accuracy and robustness of sensor data interpretation. The preprocessing phase aimed to improve the overall quality of the data. The processing phase included techniques such as normalization, data augmentation, resizing, and splitting the dataset into training, validation, and testing sets. In this research, a dataset was constructed from 300 videos, with each video containing 300 samples. Images were extracted from each frame of the videos to create an imaging dataset for deep learning algorithms. The raw dataset initially comprised 1,300 images in each class. After preprocessing, the imaging dataset was first divided into training, validation, and test subsets. In this study, the dataset was split using an 80%/10%/10% ratio for training, validation, and testing, respectively. To prevent data leakage, data augmentation was applied only to the training subset, while the validation and test sets were kept unchanged for unbiased evaluation. Data augmentation plays a crucial role in improving dataset diversity and model generalization. The augmentation process was implemented in two phases: (i) an initial augmentation stage after dataset preparation, and (ii) an additional augmentation stage after model optimization. The processing route for creating the imaging dataset is illustrated in Fig. 4, and the number of images per class after augmentation is shown in Fig. 6.

## Methods

### Traditional methods

Data from the e-nose were analyzed with different methods. Conventional analysis is a statistical and multivariate method such as principal component analysis (PCA) or linear discriminant analysis (LDA)^44^. These methods were designed as models for predicting specific leaves of plants based on features that were extracted with E-nose systems. Principal Component Analysis (PCA) is a dimensionality reduction technique frequently employed in the analysis of large datasets. This method aids in extracting valuable and actionable insights from the data. On the other hand, Linear Discriminant Analysis (LDA) works to bring samples within the same category closer together while effectively separating samples from different categories. These techniques, in the face of complex and very big data, cannot make accurate pattern recognition.

### Machine learning algorithms

Machine learning algorithms can be integrated into E-nose systems to provide automatic detection and classification techniques. Feature maps of leaf aroma were transferred to machine learning algorithms for designing predictive and recognizing models. Five machine learning algorithms, such as support vector machine (SVM), random forest (RF), decision tree (DT), k-nearest neighbor (KNN), and extreme gradient boosting (XGB).

Support Vector Machines (SVM) are an innovative approach to binary classification. They nonlinearly map input vectors to a high-dimensional feature space, constructing a linear decision boundary with strong generalization properties. Originally designed for separable data, SVM has been extended to handle non-separable cases through the use of polynomial transformations^45^. However, SVMs can be sensitive to the scaling and normalization of the data, which may necessitate careful preprocessing before modeling. Additionally, they can be prone to overfitting if the model is too complex or if the training dataset is too small.

A random forest (RF) is a type of classifier that consists of multiple tree-structured classifiers. These individual trees are combined to create a robust classification model designed to improve accuracy. Each tree in the forest votes for the most common class based on the input data^46,47^. However, random forests are not well-suited for extrapolating beyond the range of the training data. Additionally, they can be computationally expensive, particularly when working with large datasets or high-dimensional data.

A decision tree (DT) is a machine learning model that resembles a tree and is used for categorization and prediction. Each path from the root node to the leaf nodes reflects a set of categorization rules, giving it the appearance of a flowchart. Depending on whether the input features match the requirements of the paths leading to it, each leaf node is given a class. To determine the optimal spliti, deally dividing the outputs of separate classes, the root node is assessed against a number of criteria^48^. However, because minor modifications to the data can have a big effect on the model, decision trees can be unstable. They might also favor features with a greater number of missing values or features with many levels.

Among the straightforward but incredibly powerful instance-based learning algorithms is K-nearest neighbors (KNN). The algorithm uses the most suitable metrics to calculate the distance and then chooses the defined data point’s closest neighbors. The foundation of the KNN algorithm is its capacity to identify distinct clustering patterns and provide predictions as soon as it has been trained^49^. But some of the challenges of this algorithm can be pointed to computationally expensive, during the situation of handling large datasets or high-dimensional data, and sensitivity to feature scaling.

XGB is designed with two main function, regularization terms and loss function, these functions are used for preventing overfitting and predicting. One of the most effective importers for gradient boosting is XGBoost (extreme gradient boosting), which offers numerous adjustments for efficiency and output presentation. It ends up employing regularization to fight overfitting and also makes sure parallelization occurs to maximize training time. XGBoost’s collection of goals, features, and performance metrics that are tailored for different tasks is one of its traits. This algorithm is just what machine learning contests need, as it gives and delivers accuracy, speed, and versatility all at once^50^.

### Deep learning architectures

Deep learning (DL) architectures were used for recognizing various leaves of plants. One of the important methods of DL is convolutional neural network (CNN). CNN is used for image processing and high-level feature extraction. Architectures of deep learning based on hyperparameters, layers, and the number of total parameters were reported to have different accuracy. Nine popular DL architectures were used for selecting the best performance in training and test accuracy of the system. The information of deep learning architectures is shown in Table 3. After selecting best architecture, hyeprparameters of architecture should be optimized based on reducing training time, increasing response time and accuracy.

**Table 3:** Information of selected deep learning architecture.

Model	Year	Total Layers	Parameters [Million]	Top-1 Accuracy (%)	Top-5 Accuracy (%)	Refrences
AlexNet	2012	8	60	63.3	84.6	Krizhevsky et al.^51^
GoogleNet	2014	22	6.8	69.8	89.6	Szegedy et al.^52^
VGG16	2014	16	13.8	71.5	89.8	Simonyan^53^
VGG19	2014	19	14.3	71.3	89.9	Simonyan^53^
InceptionV2	2015	42	11.2	74.7	92.2	Szegedy et al.^54^
ResNet50	2015	50	25.6	76	93.0	He et al.^55^
InceptionV3	2016	48	23.8	77.9	93.7	Szegedy et al.^54^
EfficientNetB5	2019	456	30	83.6	96.7	Tan and Le^56^
EfficientNetB6	2019	518	43	84	96.8	Tan and Le^56^
EfficientNetB7	2019	620	66	84.3	97	Tan and Le^56^

### Hyperparameters

Hyperparameters have a great impact on optimizing deep learning architectures. Therefore, adjusting the best hyperparameters affects the cost of computing and training architectures. Pretraining architectures with high diversity are introduced. Deeper architecture can extract high-level features and report better accuracy, but these architectures have challenges in limited computing. Accordingly, architectures with proper accuracy were selected for optimizing hyperparameters. Parameters in preprocessing and hyperparameters in architecture both have a great impact on the accuracy of the model. In this study, after selecting the best architectures, image size, batch size, optimization, and learning rate were optimized for seeking higher performance near the decreasing rate of overfitting and computing costs.

### Fusion model

A late fusion strategy was employed to integrate the predictions of the computer vision (CV) model and the E-nose model. Late fusion is suitable for multimodal systems with heterogeneous feature spaces and different noise characteristics, as it combines model outputs at the decision level rather than directly concatenating raw features.

Let x denote an input sample and C be the number of classes (C = 26). Each modality-specific classifier produces a probability distribution over the C classes. The CV model outputs class probabilities through the Softmax layer, denoted as P_cnn_(y = c | x), while the E-nose model outputs class probabilities using the probabilistic output of the classifier (e.g., predict_proba), denoted as P_ML_(y = c | x).

Both outputs are normalized such that:

$$\sum_{C=1}^{C}p\left( y=c|x\right)=1$$

The final fused probability is computed as a weighted combination:

$$P_{{{\mathrm{final}}}} \left( {y \, = \, c \, \left| x \right.} \right) \, = \, w \, \cdot \, P_{{{\mathrm{cnn}}}} \left( {y \, = \, c \, \left| x \right.} \right) \, + \, \left( {1 - w} \right) \, \cdot \, P_{ML} \left( {y \, = \, c \, \left| x \right.} \right), 0 \, \le \, w \, \le \, 1$$

The predicted class is selected by maximizing P_final_. The fusion weight w was treated as a hyperparameter and optimized on the validation set using grid search over the range [0,1] with a step size of 0.01. For each candidate weight, the fused model performance was evaluated using Accuracy (or Macro-F1 in the case of class imbalance), and the weight yielding the best validation performance was selected.

### Evaluation model

Evaluation metrics play an important role in the deep learning and machine learning workflow, allowing us to assess the effectiveness of proposed models and gather insights on how to improve them in comparison to other existing deep learning and machine learning techniques^57^. To evaluate model performance, various metrics were used, including accuracy, precision, recall, and F1-score, which are shown in Table 4.

**Table 4:** Equation of evaluation models.

Deep learning and machine learning Equations			
$Accuracy= \frac{ (\mathrm{T}\mathrm{P}+\mathrm{T}\mathrm{N})}{ (\mathrm{T}\mathrm{P}+\mathrm{T}\mathrm{N}+\mathrm{F}\mathrm{P}+\mathrm{F}\mathrm{N})}$	(1)	$Recall=\frac{\mathrm{T}\mathrm{P}}{\mathrm{T}\mathrm{P}+\mathrm{F}\mathrm{N}}$	(2)
$precision=\frac{\mathrm{T}\mathrm{P}}{\mathrm{T}\mathrm{P}+\mathrm{F}\mathrm{P}}$	(3)	$F1-score=\frac{2\times \mathrm{T}\mathrm{P}}{2\times \mathrm{T}\mathrm{P}+\mathrm{F}\mathrm{N}+FP}$	(4)

## Results

Leaves of plants have high similarity in shape, color, texture, and odor that stretch back to the condition of foster, weather of environment, and breeds. 26 leaves were selected for integrating the E-nose sensor with the machine vision system. Data was analyzed with machine learning and deep learning algorithms. The combination of deep learning and machine learning algorithms provokes the detection of the appearance and aroma of leaves and makes it possible to also compare the performance of these platforms.

All models were trained and tested on Google Colab using an A100 GPU. Reported training and inference times were measured in this environment. While this setup allows for reproducible benchmarking, the models have not yet been deployed or tested on real edge devices. Future work will investigate deployment on web-based or mobile applications to allow real-time usage by end users.

### Developing machine learning algorithms for leaves

**Fig. 7:**
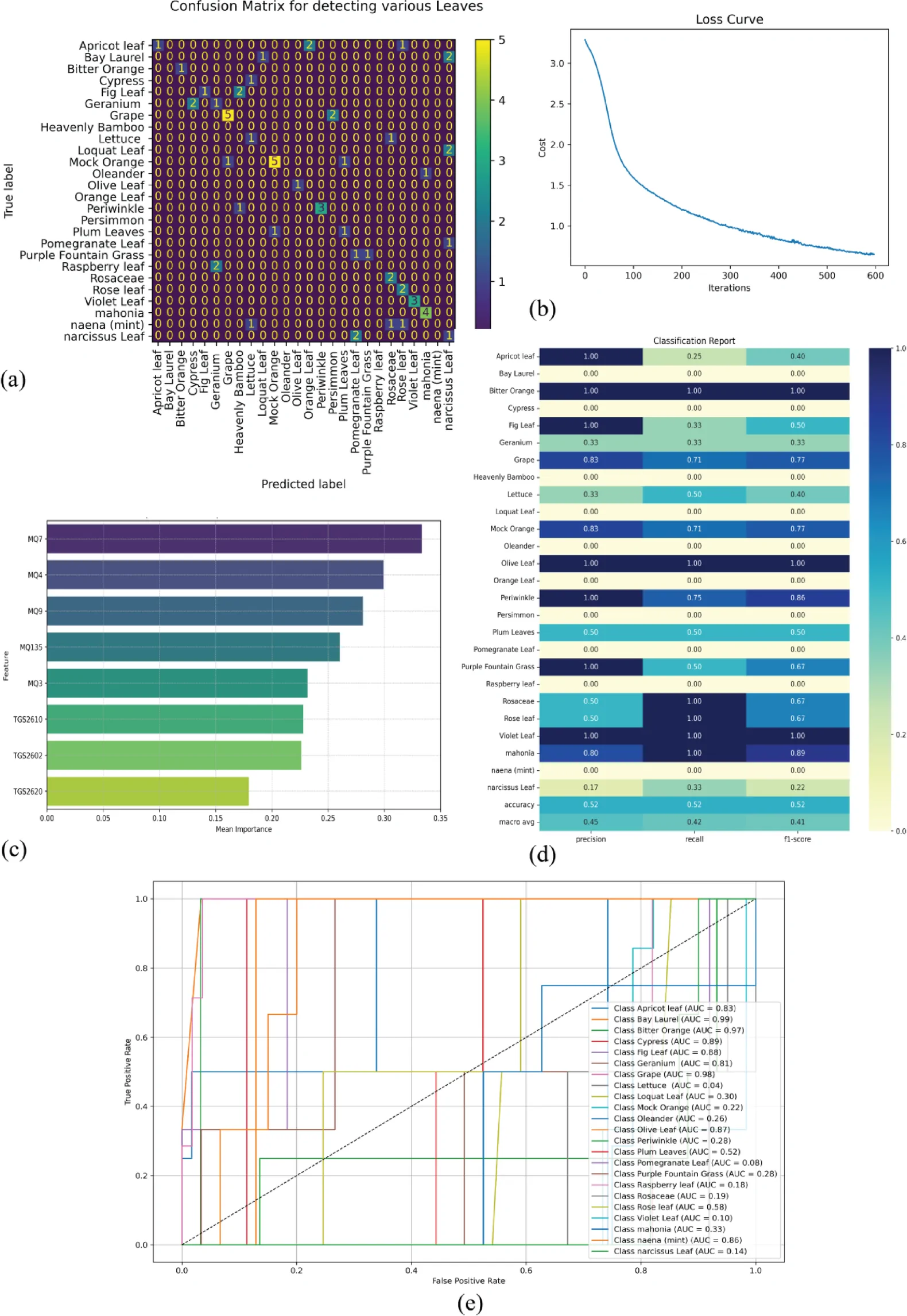
Performance of MLP5 in recognition of leaves. (**a**); Confusion matrix of MLP5. (**b**); Loss curve in 10,000 iterations of MLP5. (**c**); Feature importance of MLP5. (**d**); Classification report of each class for MLP5. (**e**); ROC curve of MLP5 in recognizing 26 classes of leaves.

The E-nose systems was susceptible to differences in the aroma of leaves. The leaves aroma of plants was analysis with 8 MOS sensors. The response of each sonsors for various samples was compeletly different. Prepared responses of E-nose were ready for analysing with pattern recognition algorithms. In first stage, multi layer precptron (MLP) with different settings were adjusted for recognizing aromas of leaves. The optimal number of neurons in the hidden layer was obtained based on a trial-and-error approach. The best architectures of MLP is illusterated in Table 4. After training and optimizing hidden layers and iterations, 5 MLP models with the highest performance compared to other models were achieved (Table 5). The arrangement and volume of neurons has a great impact on the performance of the model. The optimized layer of MLP5 was induced to achieve 52% accuracy, precision, recall, and F1-score. In spite of the fact, MLP1 has deeper neurons and the highest iteration, but the final performance was hopeless. The result of MLP5 is demonstrated in Fig. 7. Performance of MLP5 in leaf classification is shown with a confusion matrix in Fig. 7a. The decreasing loss curve throughout the iterations in Fig. 7b illustrated the convergence of the model in minimizing the loss of the model for the higher performance. The impact of sensors in extracting the best feature and creating feature maps with models is shown in Fig. 7c. The MQ7 sensor had the highest impact in creating a feature map based on measuring permutation importance. The classification report of MLP5 with precision, recall, and F1-score for each class is shown with a heatmap in Fig. 7d. The area under the curve (AUC) of the MLP5 for each class of leaves is displayed in Fig. 7e.

**Table 5:** Performance of best artificial neural network architectures in training process.

Model	Hidden layer size	Active-iteration	Accuracy (%)	Precision (%)	Recall (%)	F1-Score (%)
MLP1	(100, 80, 60, 40, 30)	2000-ReLU	37	38	37	35
MLP2	(100, 80, 60, 40, 30)	10,000-ReLU	37	36	37	37
MLP3	(80, 60, 40, 30, 26)	10,000-ReLU	41	40	38	35
MLP4	(50, 40, 30, 26)	10,000-ReLU	48	47	48	44
MLP5	(60, 40, 30, 26)	10,000-ReLU	52	52	52	52

The relatively low performance of the MLP models (best accuracy ≈ 52%) can be explained by the characteristics of the E-nose dataset and the model structure. The extracted E-nose features are low-dimensional, highly correlated across MOS sensors, and can be affected by sensor drift and environmental variations. In such conditions, distance-based or margin-based classifiers (e.g., KNN and SVM) often provide better generalization than MLP, especially when the number of samples per class is limited and the decision boundaries are not strongly nonlinear. Moreover, MLP models are sensitive to hyperparameter settings (learning rate, hidden neurons, regularization) and may require larger datasets or more discriminative feature representations to achieve competitive performance. Therefore, the MLP results indicate that the E-nose feature space is better captured by classical ML classifiers in this study, while the deep learning models demonstrate their strength mainly in the computer vision modality.

**Fig. 8:**
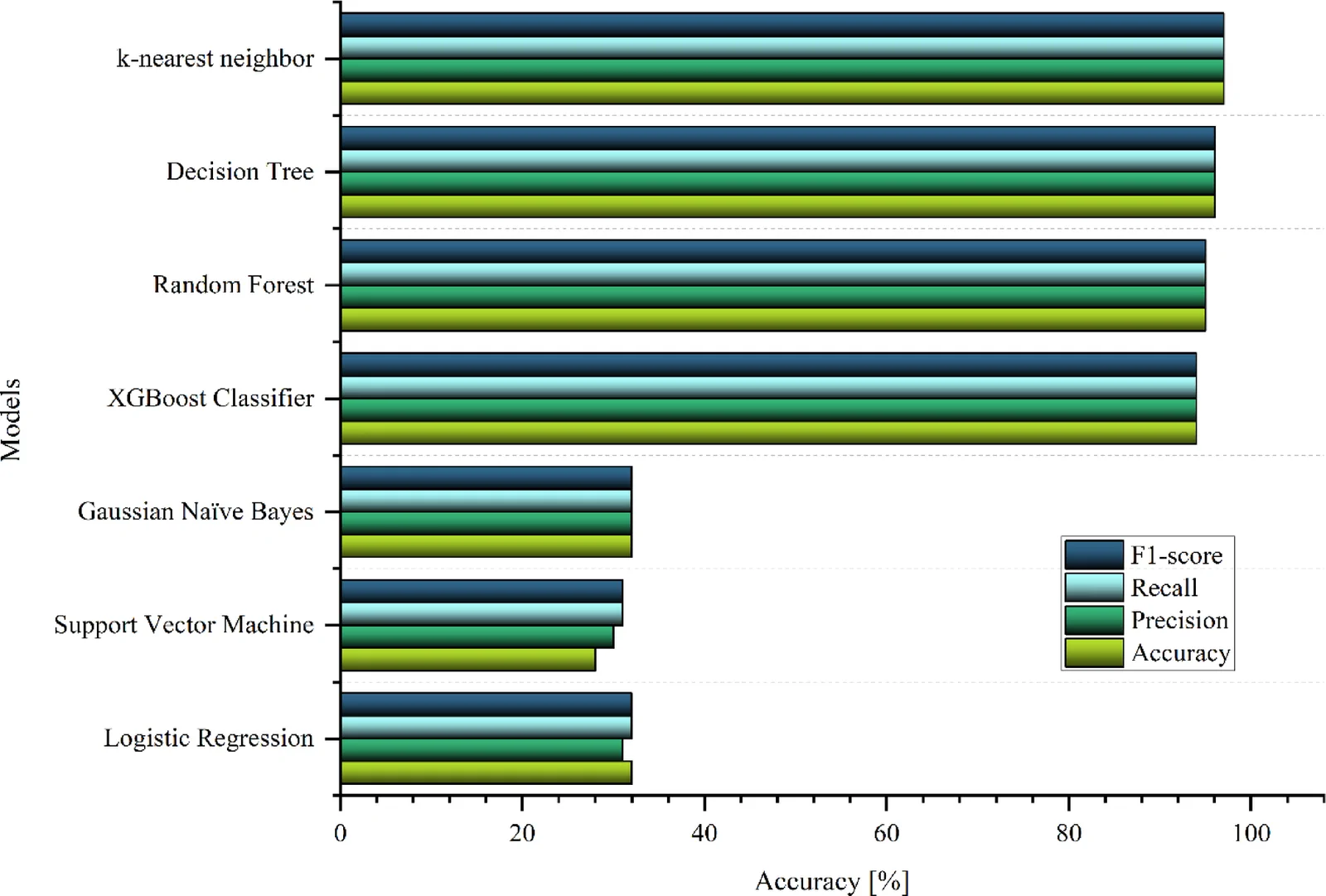
Comparison performance of machine learning algorithms.

**Fig. 9:**
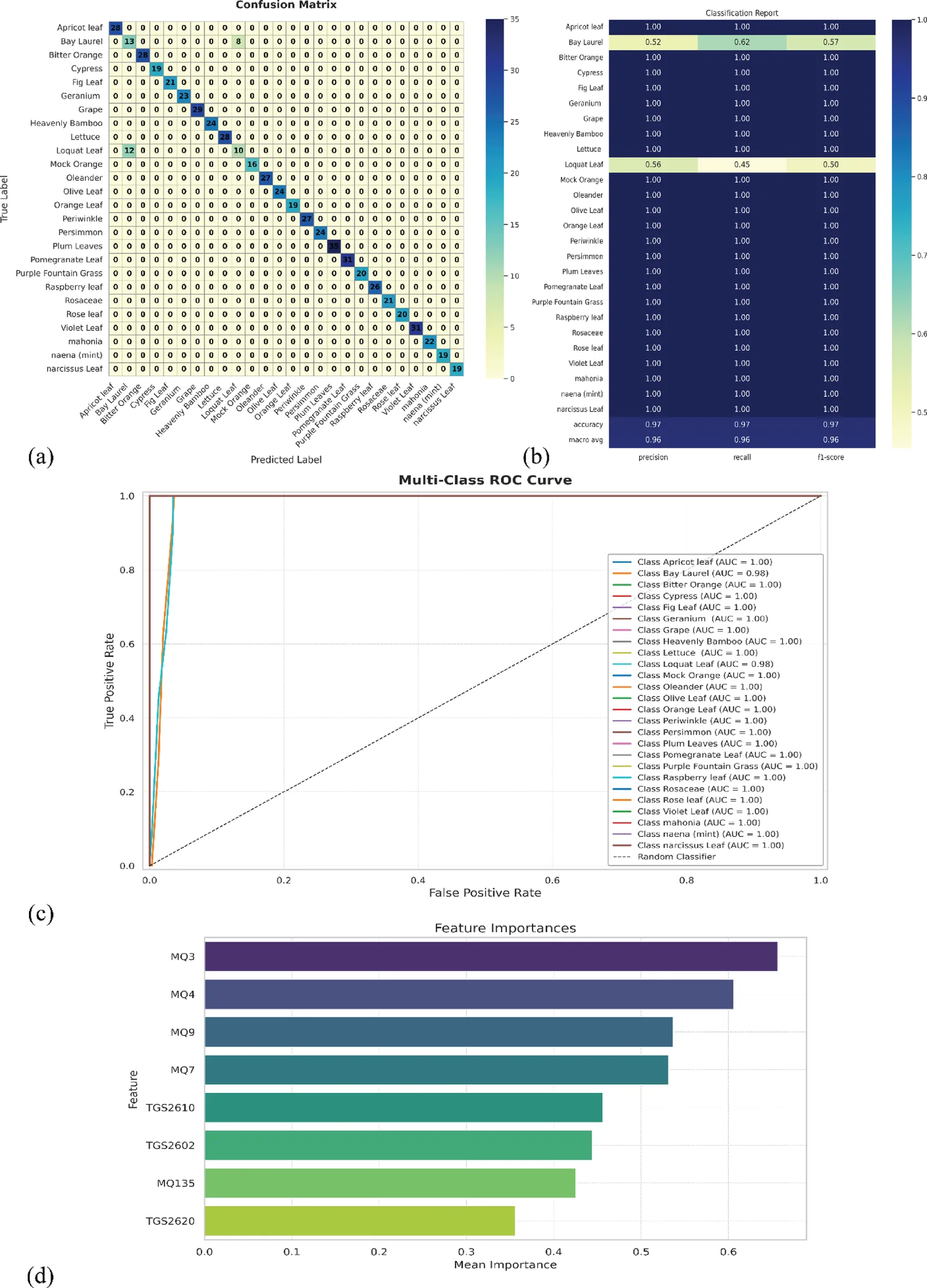
Performance of KNN in recognition of leaves. (**a**); Confusion matrix of KNN. (**b**); Classification report of each class for MLP5. (**c**); ROC curve of MLP5 in recognizing 26 classes of leaves. (**d**); Feature importance of KNN.

Machine learning algorithms such as support vector machine (SVM), naïve bayes (NB), extreme gradient boosting (XGB), decision tree (DT), random forest (RF), k-nearest neighbor (KNN), and logistic regression (LR) were implemented for boosting the performance of the model in detecting compared MLPs. The information and performance of models are reported in Table 6. The settings of each model were obtained after more training on the prepared data set. The compared performance of 7 machine learning algorithms and MLPs demonstrated that KNN was the best model for recognizing leaves based on E-nose feature maps that are shown in Fig. 8. Leaves from 26 plant species attained 97% classification accuracy by the KNN algorithm. Detailed performance assessment of the KNN model is provided in Fig. 9. The performance of KNN in identifying leaves is shown with a confusion matrix in Fig. 9a. The classification report of KNN showed the classes of “bay laurel” and “loquat leaf” were recognized with the least accuracy in Fig. 9b. The receiver operating characteristic (ROC) curve of the KNN model is presented in Fig. 9c. Eventually, sensor importance analysis for feature extraction highlighting the most influential sensors in the classification process is shown in Fig. 9d. In the next step, the deep learning algorithms and their corresponding hyperparameters are initialized for evaluating and recognizing various species of leaves based on appearance features.

**Table 6:** Optimal values from hyperparameters in machine learning algorithms.

Model	Parameters	Train ACC (%)	Test ACC (%)
Logistic regression	penalty: l2, C: 1.0, Solver: lbfgs, Max_iter: 100, Multi_Class: multinomial	33	32
Support vector Machine	Kernel: rbf, C: 2, Gamma: 1, Kernel: Sigmoid	31	28
Gaussian Naïve bayes	var_smoothing = 1e-9	33	32
XGBoost classifier	N_estimators: 100, Max_depth: 10, Min_samples_split:4	95	94
Random forest	N_estimators: 100, Max_depth: 70, Min_samples_split:4	95	95
Decision tree	Max_depth: 50, Min_samples_split:2	96	95
k-nearest neighbor	n_neighbors: 8, weights: uniform, Algorithm: BallTree	97	97

### Optimizing deep learning algorithms

After tracking and identifying the aroma of leaves, 6 popular new deep learning and vision transformer algorithms were applied to recognizing and extracting appearance feature maps. Initially, 6 deep learning algorithms with the initial settings were implemented, and the information of the models is reported in Table 7.

**Table 7:** Performance of deep learning architectures on raw data for classifying six quality categories of different saffron species, with adjusted and base hyperparameters as needed.

Architecture	Epochs	Image size	Learning rate	Batch Size	Optimizer	Training Loss	Training ACC (%)
EfficientNetV2B0	50	128 × 128	10^–2^	32	SGD	1.23	45
MobileNetV3	50	128 × 128	10^–2^	32	SGD	1.11	40
ResNet50	50	128 × 128	10^–2^	32	SGD	0.95	50
ConvNeXtTiny	50	128 × 128	10^–2^	32	SGD	0.88	43
EfficientNetB7	50	128 × 128	10^–2^	32	SGD	0.7	80
InceptionV3	50	128 × 128	10^–2^	32	SGD	0.502	78

**Fig. 10:**
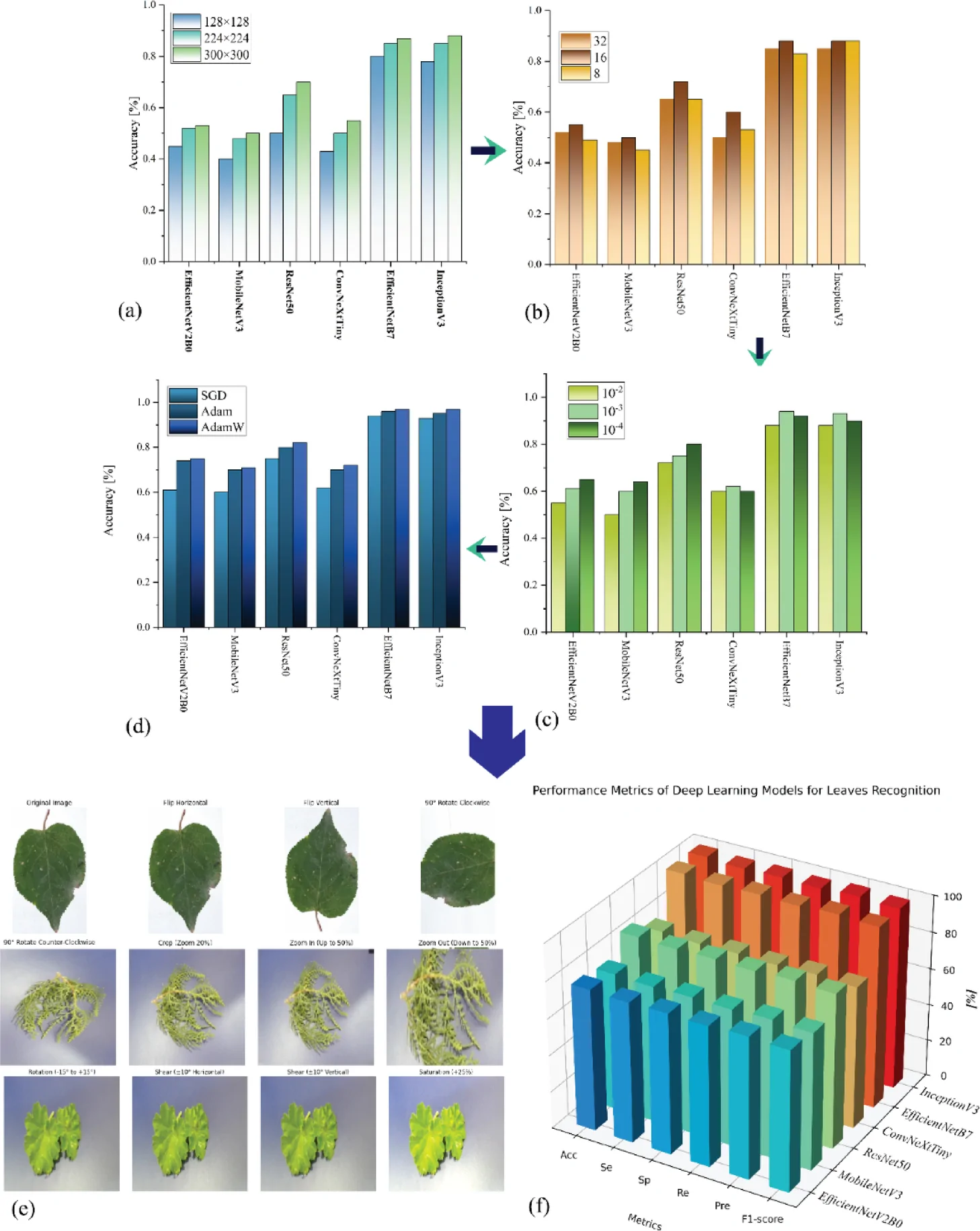
Demonstrated workflow of optimizing hyperparameters and impact on performance of models. (**a**); impact of image size on the accuracy of models. (**b**); effect of batch size on performance of models. (**c**); impact of different learning rates on the accuracy of models. (**d**); analyzing different optimizers on models. (**e**); Some of the implemented data augmentation techniques. (**f**); performance of optimized models and hyperparameters.

To clarify the performance improvement from the initial results (Table 7) to the final accuracy (~ 99%), the optimization was performed in a step-wise manner. Table 7 reports the baseline performance using raw images and default hyperparameter settings. Then, the effect of key factors (image size, batch size, learning rate, optimizer selection, and data augmentation) was evaluated sequentially, and the accuracy gain at each stage is reported in Fig. 10(a–f). Therefore, the final high accuracy is not due to a single change, but rather the cumulative effect of systematic hyperparameter tuning and dataset enhancement. (Table 8).

**Table 8:** Comparison performance of models.

Model	Accuracy (%)	Recall (%)	F1-score (%)	Response time [ms]
KNN	97	97	97	5
InceptionV3	99.5	99.4	99.2	8
Fusion model	99.8	99.7	99.6	10

The performance of deep learning and vision transformer algorithms depended on adjusting the layer, the volume of the model, and optimizing hyperparameters. First, the best algorithms based on accuracy and loss were determined for focusing and optimizing hyperparameters and models. EfficientNetB7 and InceptionV3 had better performance comparisons than other algorithms. But always higher accuracy and least loss have costs in implementing the model.Costs involved high training time, which was about 10,800 and 8820 s, and response time was about 20 and 18 ms. Decreasing computing costs and preserving performance is an informative stage in designing optimized algorithms.

Optimizing parameters is started by adjusting image size. Increasing image size has a direct impact on the accuracy of the model because it permits deeper algorithms for extracting high-level features. In spite of the fact, too high an image size induces high computing costs. Therefore, the image data set was synchronized with three image sizes (128 × 128, 224 × 224, and 300 × 300) for evaluating the performance of models. Performance of InceptionV3 and EfficientNetB7 was boosted in each step of increasing image size that is shown in Fig. 10a. But an image size of 224 × 224 creates a balance between accuracy and computing costs. The accuracy of InceptionV3 and EfficientNetB7 increased from 78 and 80% to 85%.

Updating weights and usage of memory and GPU was dependent on batch size. Balanced batch size induced normal computing time and acceptable accuracy and loss for algorithms. After the previous update, algorithms were implemented with 3 batch sizes (32, 16, 8) for assessment performance. The performance of InceptionV3 and EfficientNetB7 with a batch size of 16 was increased by about 3%, as demonstrated in Fig. 10b.

Amount of learning rate direct connected to convergence rate, fluctuation during training and impact on accuracy of models. Three learning rate contain (10^–2^, 10^–3^ and 10^–4^) were executed, decreasing learning rate had positive impact on accuracy of models that is shown in Fig. 10c. But learning rate of 10^–4^ caused more fluctuation and overfitting during process. Under the optimal learning rate settings, InceptionV3 and EfficientNetB7 achieved classification accuracies of 93% and 94%, respectively, in recognizing leaf species.

**Fig. 11:**
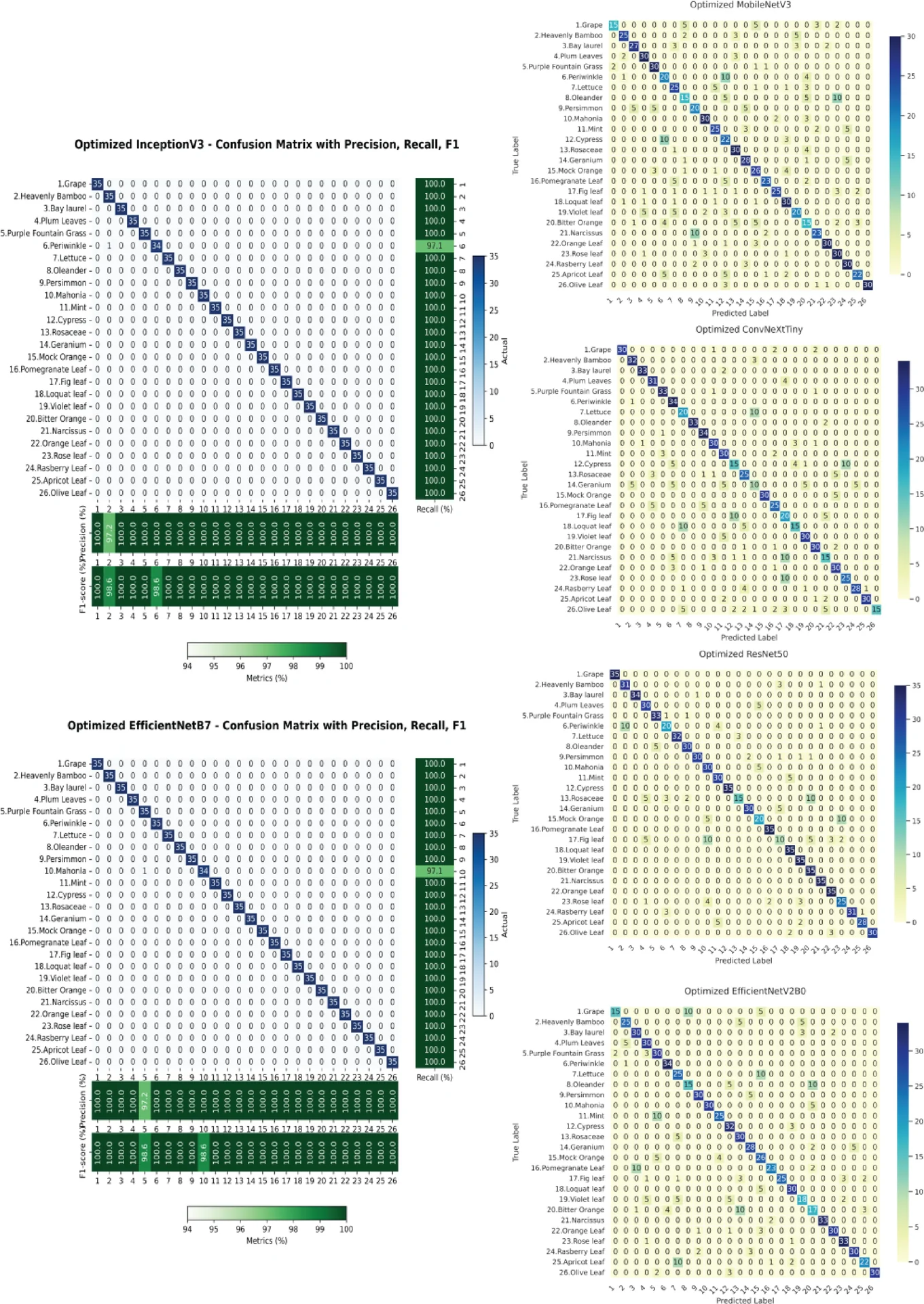
Confusion matrix of the six deep learning algorithms in recognizing 26 species of leaves.

The final informative stage in adjusting the model was determining optimizers. The impact of 4 optimizers, such as SGD, Adam, and AdamW, was analyzed during the training process for deep learning algorithms that is illustrated in Fig. 10d. The performance of InceptionV3 and EfficientNetB7 with AdamW after 15 epochs didn’t change, and the highest accuracy that was reported was 97%. Optimizing hyperparameters induced increasing performance of InceptionV3 and EfficientNetB7. Another way to increase performance is to boost the data set. Data augmentation was implemented in two phases: the first immediately after data preprocessing and the second following model optimization. Dataset diversity was increased to 156,000 images by using various techniques, including horizontal and vertical flipping, rotation at three angles, cropping, zooming in and out, saturation adjustment, and shearing. Some of the data augmentation techniques that were implemented are shown in Fig. 10e. A satisfactory data set caused a positive impact in increasing the accuracy of models, such that the performance of models, such as specificity, sensitivity, recall, accuracy, and F1 score, is deeply in Fig. 10f. Subsequently, InceptionV3 and EfficientNetB7 could recognize 26 leaf species with 99% accuracy. The corresponding confusion matrices for the models are shown in Fig. 11.

**Fig. 12:**
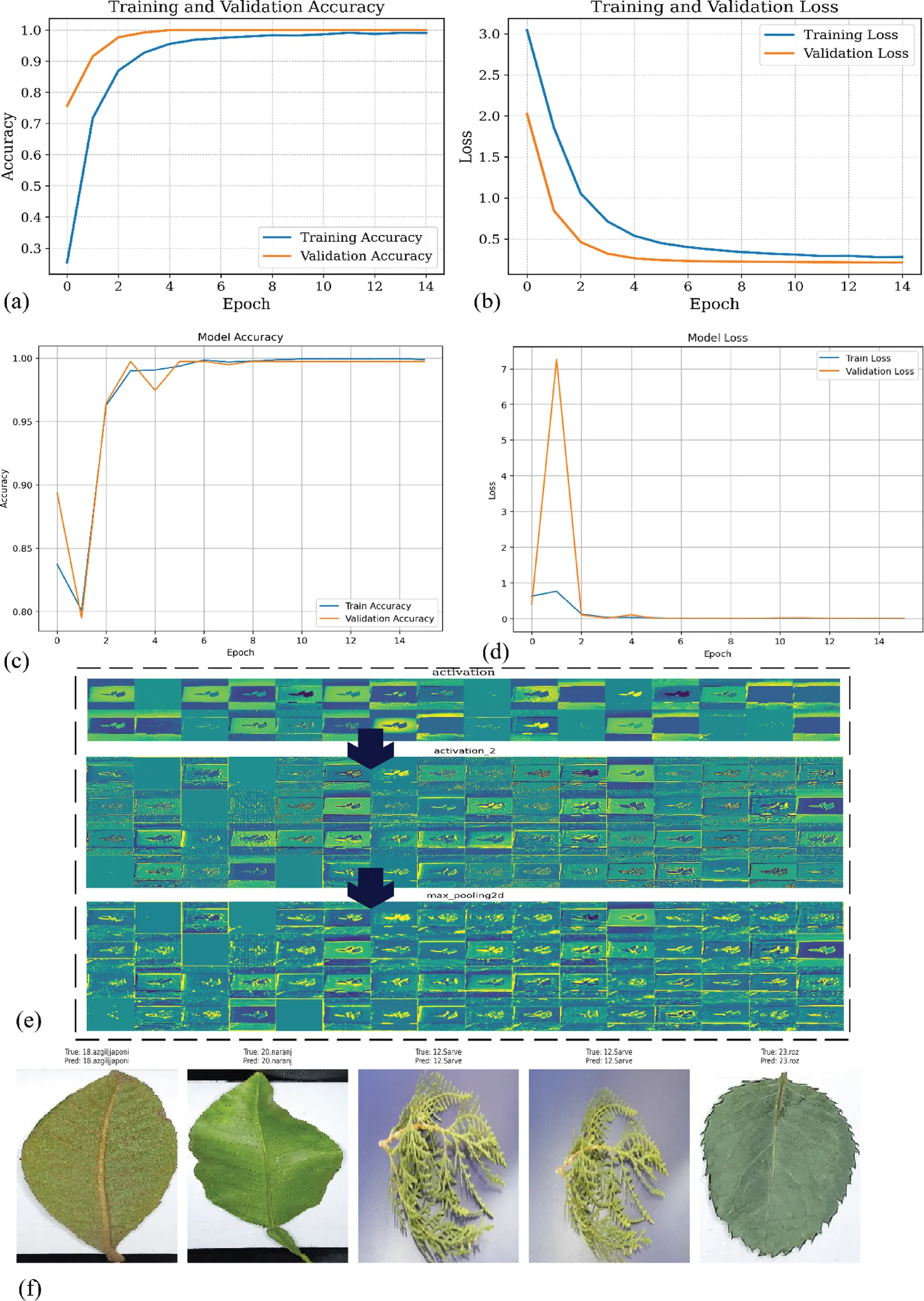
Comparative analysis of training and loss process of InceptionV3 and EfficientNetB7. (**a**); training process of InceptionV3. (**b**); loss process of InceptionV3. (**c**); training curve of efficientNetB7. (**d**); loss curve of EfficientNetB7. (**e**); performance of the activation and max pooling layer of InceptionV3 in extracting features from leaves. (**f**); performance of the model in recognizing different leaves with Inceptionv3.

**Fig. 13:**
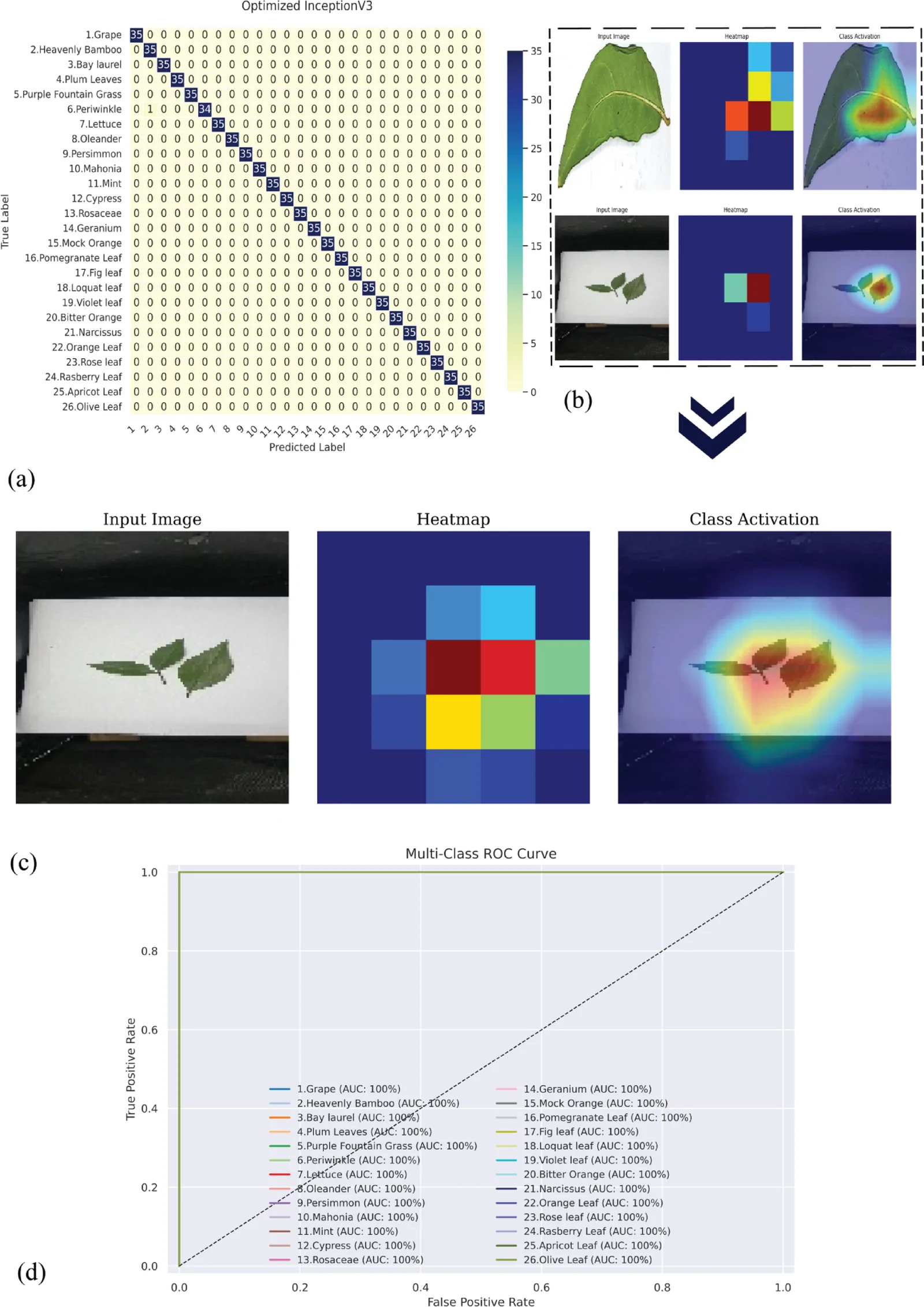
Performance of InceptionV3 and comparison region detection for the feature in EfficientNet and InceptionV3. (**a**); Confusion matrix of InceptionV3 for detecting 26 species of leaves. (**b**); Heat map class activation method of EfficientNetB7. (**c**); Heat map feature extraction method of InceptionV3. (**d**); ROC curve of InceptionV3 for the detection of different leaves.

After analyzing the results of the models, InceptionV3 and EfficientNetB7 have the same performance, but with massive differences in training time and response time. After final adjusting and optimizing, InceptionV3 and EfficientNetB7 had 7200 and 8280 s of training time and 10 and 8 ms of response time. The training and loss process of InceptionV3 is shown in Fig. 12a and b. The training and loss process of EfficientNetB7 is displayed in Fig. 12c and d. Analyzing the developing process and computing costs showed InceptionV3 had the least fluctuation in process, better training time, and response time. The performance of some layer in InceptionV3 in feature extraction is shown in Fig. 12e. The result of testing models in detecting some of the classes is depicted in Fig. 12f. The result of InceptionV3 is shown with a Confusion matrix and the receiver operating characteristic (ROC) in Fig. 13a and d.

InceptionV3 had better performance in the detection region of feature extraction and detecting leaves comparison than EfficientNetB7, as shown in Fig. 13c. The region of feature extraction in EfficientNetB7 is shown in Fig. 13b. InceptionV3, in contrast to EfficientNetB7, focused on full sample size and could extract more high-level features and better performance in real time.

### Hybrid model fusion of E-nose and computer vision based deep and machine learning

Integrating computer vision and E-nose systems was a crucial and novel stage of designing a framework. The fusion model analyzes both the aroma and appearance of 26 varieties of leaves in multiple situations. Hybrid algorithms were induced to cover error crisis, robust confidentiality, and stability in unstable conditions, preserving the performance of the model in a sample with a light amount of data, and showing a confidential real-time processing method. Separate models, InceptionV3 with 99% accuracy in analyzing vision and KNN with 97% accuracy in analyzing aroma, had spectacular performance. But for preventing future errors in related situations and missing data, and to attract the opinion and trust of the user, late fusion was designed for integrating the output of models and making a final decision. Late fusion framework as follows;

X = Sample or leaves

C = Number of categories (26)

Each model produces a probability distribution over 26 classes with “Softmax” and “predict_proba”, where the sum of probabilities equals to one.

$$Inception\;V3\;output:(x/c = P_{cnn} \left( {y\sum\limits_{(C = 1)}^{C} {c\;[1,0]} \in Pcnn\;(x)} \right) = 1$$

$$KNN\;output:(x/c = P_{knn} \left( {y\sum\limits_{(C = 1)}^{C} {c[1,0]} \;P_{knn} (x)} \right) = 1$$

After normalization out put of model as follows:

Both outputs are normalized such that $\sum_{C=1}^{C}p\left( y=c|x\right)=1$

$${\text{Hybrid model decision: }}P_{knn} \times \left( {w - 1} \right) \, + \, P_{cnn} \times w \, = \, P_{final} , \, w \in \left[ {0,1} \right]$$

The optimal weight w was selected on a validation set by performing grid search in the range [0,1] with a step size of 0.01, maximizing classification accuracy or Macro-F1 in the case of imbalanced data.

After validating out put of model selecting best weight for late fusion model based on accuracy:

$${\text{Final Fusion model}}:P_{knn} \times 0.258 + \, P_{cnn} \times 0.742 \, = \, P_{final}$$

High weight of P_cnn_ indicates that the vision-based CNN features were more discriminative, while the E-nose contributed complementary information for better robustness.

## Discussion

Optimizing the model to increase performance is one of the important stages of developing a framework. Follow that, optimizing VGG16 and AlexNet by appending specific layers showed a positive impact^58^, while improving architecture with integrating specific layers to ResNet18 yielded notable results^59^. Similarly, robust algorithms have been developed by feeding different blocks of data sets that are specific^60^. Beyond optimization architectures, developing multi-sensor systems has also been described behind optimizing algorithms to exploit full feature extraction^61^. Most previous studies have encountered challenges in selecting the method of integration or have focused on optimizing specific hyperparameters. In addition, in most investigations, feature extraction was typically performed by either processing the two methods separately or by combining their outputs only after the extraction stage, rather than integrating them within the modeling process.

In this study, a multi-sensor system, a progressed multiproposed data set was merged with optimized deep learning and machine learning algorithms to design a fusion model. Ensuring sufficient diversity in the dataset is one of the big challenge in developing deep learning and machine learning algorithms. This issue was covered by integrating the image and aroma datasets. The important trait of the dataset was gathered during 2 years from different tree locations and seasons. After preprocessing methods on raw data set, two phases of data augmentation techniques were implemented for increasing the diversity of the datasets. A powerful data set required improved algorithms for analysis. Accordingly, MLPs and 7 machine learning models were optimized and implemented for recognizing leaves based on the E-nose system. In the next step, six deep learning techniques were presented to optimize hyperparameters for designing a fast and accurate intelligent framework. In the final step, a fusion model of E-nose and computer vision by deep and machine learning algorithms induced synergy in recognizing was designed. The fusion model is more usable because the model can identify and cover the missing data comparisons of different single methods.

## Conclusion

Designing a recognition system for plant leaves is crucial for preserving endangered species, protecting biodiversity, and enabling accurate management of pests and diseases affecting plants. Subsequently, an E-nose with 8 sensors and an adjusted computer vision platform were designed for accumulating data. The massive dataset of 26 species of leaves, which was gathered during 2 years in different locations, trees, and seasons, consisted of 11,857 samples. The initial processing was induced on a decreasing dataset to 7800 leaves. Then, a preprocessing method for E-nose and computer vision data was implemented, and a new data set for both consisted of 15,600 data points. Data augmentation techniques were implemented in two phases to increase the image dataset to 156,000 images. After that, MLPs and machine learning algorithms were adjusted and optimized based on the E-nose data set. KNN algorithms were the best algorithms compared to others, with 97% accuracy. Meanwhile, 6 deep learning algorithms were implemented, and hyperparameters such as batch size, learning rate, image size, and optimizer were optimized based on performance. InceptionV3 could recognize 26 leaves based on appearance with 99% accuracy. To overcome the limitations of using each modality separately, the fusion model of the E-nose system and computer vision with deep learning and machine learning was designed. The proposed model could recognize 26 species of leaves with 99.8% accuracy and a 10-ms response time per sample, surpassing the performance of individual models. The fusion model was designed to cover the limitations of models in highly similar, missing data and to increase accuracy and robustness. Therefore, the hybrid platform demonstrates strong potential as a reliable, scalable, and fast recognition tool for agricultural applications and biodiversity conservation.

### Significance and practical implications

The proposed multisensory framework demonstrates that combining olfactory information captured by an electronic nose with visual information extracted from leaf images can substantially improve plant leaf recognition accuracy, especially in scenarios where single-modality systems are unreliable due to high inter-class similarity. This work contributes to the plant recognition literature by providing a comprehensive benchmark of machine learning, ANN, and deep learning models on a real multispecies dataset, and by introducing a decision-level late fusion strategy that enhances reliability and robustness. From a practical perspective, the proposed framework can support agricultural monitoring, medicinal plant identification, biodiversity conservation, and rapid screening applications, where fast and accurate recognition is required and expert knowledge may not be available.

### Limitations

Despite the promising results, several limitations should be acknowledged. First, the dataset covers 26 plant species that were selected based on availability and importance; therefore, although the dataset is diverse, it does not represent the full botanical diversity of plant species. Second, while the data were collected across multiple locations and over an extended period, environmental factors such as illumination variability in images and humidity/temperature effects on E-nose signals may still influence model behavior. Third, the late fusion weighting factor was optimized for the current dataset and experimental conditions; consequently, direct transfer of the same weight values to other datasets may not yield optimal performance. Finally, although inference time was reported using Google Colab hardware (A100 GPU), real-time deployment on embedded/edge devices was not evaluated in this study.

### Future work

Future research will focus on extending the dataset by incorporating more plant species, additional seasonal conditions, and broader environmental variability to further improve generalization. Moreover, robustness experiments will be conducted under missing-modality scenarios (e.g., partial failure of E-nose sensors or unavailable image modality) to evaluate real-world reliability. Another important direction is to explore alternative fusion strategies such as feature-level fusion, attention-based fusion, and adaptive weighting mechanisms that automatically adjust modality contributions depending on confidence and data quality. Finally, deployment of the proposed framework on lightweight edge platforms and mobile/web-based systems will be investigated to enable real-time plant recognition applications for end users.

## Data Availability

The data that support the findings of this study are available from the corresponding author upon reasonable request.
